# Explainable gait recognition with prototyping encoder–decoder

**DOI:** 10.1371/journal.pone.0264783

**Published:** 2022-03-11

**Authors:** Jucheol Moon, Yong-Min Shin, Jin-Duk Park, Nelson Hebert Minaya, Won-Yong Shin, Sang-Il Choi

**Affiliations:** 1 Department of Computer Engineering and Computer Science, California State University, Long Beach, CA, United States of America; 2 School of Mathematics and Computing (Computational Science and Engineering), Yonsei University, Seoul, Republic of Korea; 3 Department of Computer Science and Engineering, Dankook University, Yongin-si, Gyeonggi-do, Republic of Korea; Vellore Institute of Technology: VIT University, INDIA

## Abstract

Human gait is a unique behavioral characteristic that can be used to recognize individuals. Collecting gait information widely by the means of wearable devices and recognizing people by the data has become a topic of research. While most prior studies collected gait information using inertial measurement units, we gather the data from 40 people using insoles, including pressure sensors, and precisely identify the gait phases from the long time series using the pressure data. In terms of recognizing people, there have been a few recent studies on neural network-based approaches for solving the open set gait recognition problem using wearable devices. Typically, these approaches determine decision boundaries in the latent space with a limited number of samples. Motivated by the fact that such methods are sensitive to the values of hyper-parameters, as our first contribution, we propose a new network model that is less sensitive to changes in the values using a new *prototyping encoder–decoder* network architecture. As our second contribution, to overcome the inherent limitations due to the lack of transparency and interpretability of neural networks, we propose a new module that enables us to analyze which part of the input is relevant to the overall recognition performance using *explainable* tools such as sensitivity analysis (SA) and layer-wise relevance propagation (LRP).

## 1 Introduction

### 1.1 Background

Human gait, i.e., the way in which people walk, is sufficiently unique to distinguish one individual from another. Gait information has been utilized for diverse applications such as disease diagnosis [1] and biometric authentication [2]. Compared to other biometric authentication methods, gait recognition is advantageous in that it is robust against impersonation attacks, not necessarily requiring vision sensors or physical contacts with sensing devices to collect data [3].

A gait recognition framework comprises two core components: data acquisition devices and data analysis algorithms. It captures representational data of the gait and identifies individuals by classifying such data using different algorithms. To be more precise, the gait information can be captured by using vision sensors, pressure sensors, and inertial measurement units (IMU); then, the captured data are classified using the linear discriminant analysis (LDA), *k*-nearest neighbor (*k*-NN), hidden markov model (HMM), support vector machine (SVM), convolutional neural network (CNN), or the combinations thereof [3]. A recognition problem can be categorized into two types: the closed set problem and the open set problem. Whereas the closed set recognition tests samples of classes known from training, the open set recognition deals with incomplete knowledge given at the time of training and tests not only known but also unknown classes [4], which is a more challenging task. While the majority of gait recognition frameworks have focused on the closed set recognition, few approaches have addressed the open set recognition in the literature [5].

### 1.2 Main contributions

**Fig 1:**
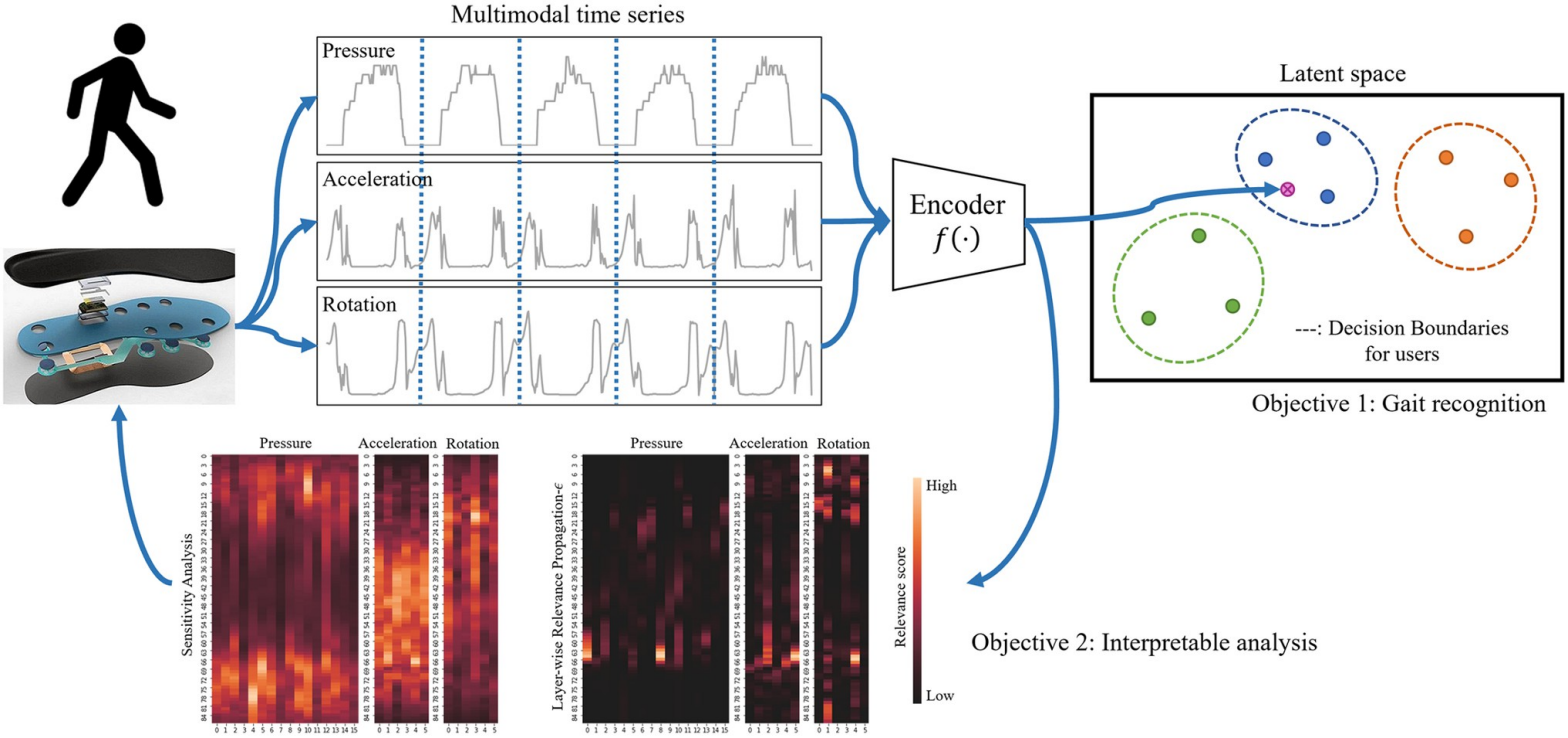
Illustration of the motivation and the objectives. Our study is to recognize a set of users from their gait patterns using a encoder network and to provide an interpretable analysis of the network using the XAI method.

Fig 1 shows the motivation of the objectives of our study. We assume a wireless environment in which all participants wear shoes with sensor-equipped insoles that can communicate wirelessly. This is due to not only an ease of data acquisition but also an availability of high-quality sensors. Under such a circumstance, as an aspect of data acquisition, the time series of each individual’s gait is captured by pressure sensors, a 3D-axis accelerometer, and a 3D-axis gyroscope installed in the insoles of the participants’ shoes. Because the data are collected by the insoles, different than other publicly available datasets [6, 7], pressure values between the foot and the ground can be measured in addition to the IMUs. Using the pressure values, the continuous gait data are segmented into separate unit steps for the gait recognition framework to perform in a more efficient and effective manner along with the human walking cycles [8], which consist of a stance phase and a swing phase [9]; the stance phase is the time when a foot is on the ground, and the swing phase is the entire time when a foot is in the air. Using the fact that the pressure values should be zero during the swing phase, the continuous data are split into unit steps. When forming unit steps, Gaussian smoothing is applied to reduce potential errors [8] such that pressure sensors sporadically show non-zeros during the swing phase [10]. To utilize the merit of using pressure values, we collected the data from 40 participants using the insoles instead of using public database contains only acceleration and gyroscope data.

As our first main contribution, we propose an encoder–decoder network model with multiple 1D convolutional layers. The encoder maps the multimodal unit steps into embedding vectors in a latent space, and the decoder reconstructs the unit steps from the embedding vectors. To train this network, a linear combination of two loss functions is used, i.e., *L* = *L*_*triplet*_ + λ*L*_*proto*_ where λ ≥ 0. Here, the first loss function, denoted by *L*_*triplet*_, is based on the triplet loss [11], and the second loss function, denoted by *L*_*proto*_, similarly follows that of the denoising autoencoder [12]. The *L*_*triplet*_ widens the distance between the embedding vectors of the heterogeneous unit steps while narrowing the distance between the embedding vectors of the homogeneous unit steps in the latent space. For homogeneous unit steps, we compute their prototype by averaging out over the unit step data; then, the *L*_*proto*_ forces the encoder–decoder network to minimize the difference between the homogeneous unit steps and their prototype. To develop and evaluate our gait recognition system to effectively address an under-explored *open set* recognition problem, we split the data into *training*, *known test*, and *unknown test* sets.

Once the encoder–decoder network is trained for the embedding vectors, the one-class support vector machine (OSVM) [13] is used to classify the unit steps. A *few* unit steps of each person in the *known test* set are randomly selected. Using the embedding vectors of the selected unit steps, OSVM is trained to compute a decision boundary for each subjects in the *known test* set. The classifiers are thereby capable of identifying whether a unit step belongs to any of the known classes. During the test phase of the system, when an unseen unit step of a subject is given, it is first mapped into an embedding vector using the encoder. The embedded vector is then examined if it is within the decision boundary of the class whose centroid is the closest among the known classes. Otherwise, it is rejected.

On the other hand, it is worth noting that the use of such neural network-based models has generally been regarded as a black-box because of the lack of transparency and interpretability [14]. The highly non-linear nature of neural networks hinders our attempt to understand the decision-making process of such models. To overcome this barrier, studies on *explainable* artificial intelligence (XAI) have emerged [15, 16], allowing transparency and interpretability to be improved in neural networks. Improved transparency offers context to the model decision, which thus leads to several benefits. First, XAI can build trust for users of a given model. Second, the interpretation itself can be used as extra information to obtain a more complete understanding [14].

The application of XAI for gait data analysis is still largely under-explored despite its potential. Several recent studies have applied XAI to the closed set gait recognition problem [17, 18]. Our study aims to utilize XAI to understand the process of the open set gait recognition. In this study, as our second main contribution, we incorporate two well-known XAI tools, namely sensitivity analysis (SA) [19] and layer-wise relevance propagation (LRP) [20], into our gait recognition framework. Each method calculates *attribution maps*, where the values indicate the importance of the input when the underlying model returns the output.

To interpret the encoder, we apply SA and LRP to the embedding vectors. However, unlike the closed set recognition models, we take the expectation of all attribution maps calculated from each dimension of the embedding space because the embedding space has no explicit interpretation. Finally, by averaging out the attribution maps over all training subjects, it is possible to obtain a common attribution map that represents important parts of the entire *training* set for gait recognition.

The main contributions of this study are summarized as follows:

- Using a combination of the triplet and prototype loss functions allows our encoder–decoder network to be more robust and less dependent on the values of hyper-parameters;
- XAI approaches are shown to obtain insights from a human interpretable analysis of a neural network-based open set gait recognition model, which demonstrates how high and low relevant parts of the data affect the accuracy of the recognition.

The remainder of this paper is organized as follows. In Section 2, we summarize significant studies that are related to our work. In Section 3, we describe the dataset for gait recognition. Section 4 explains our proposed methods. Experimental results are provided in Section 5. Finally, we summarize the paper with some concluding remarks in Section 6.

## 2 Related work

The method that we propose in this paper is related to two broader areas of research, namely gait recognition and explainable neural networks.

### 2.1 Gait recognition

The use of vision sensors led to the beginning of gait recognition analyses [21]; follow-up studies have actively been carried out in the literature [22, 23]. Despite challenging conditions for collecting data in vision-based recognition (e.g., requiring only the subject of interest in the video sequences), the accuracy of gait recognition based on these vision-based approaches is insufficiently high and yet unstable depending on the viewpoint and orientation of the sensing devices [24]. To overcome these obstacles, not only subjects in video sequences were segmented and individually tracked [24], but also 3D construction and view transformation models were used [25]. A view-adaptive mapping approach for gait recognition was also developed in [26] to alleviate the free-view gait recognition problem in which the view angle is often unknown, dynamically changing, or does not belong to any predefined views.

In addition to such vision-based approaches, pressure sensors and IMUs have been broadly used to collect data in recent gait recognition analyses. IMUs typically consist of an accelerometer, a gyroscope, and a magnetometer. For example, gait information was gathered from IMUs attached to multiple parts of each participant’s body [27], and then the individuals were identified using a CNN-based predictive model [28]. Later, pressure sensors and IMUs installed in wearable devices, e.g., smartphones, fitness trackers, or shoe insoles [29], were used. In a study on smartphone-based gait recognition [30], data from IMUs in smartphones were analyzed using a mixed model of CNN and SVM [31]. In another study, null space LDA was applied to analyze gait data from pressure sensors and accelerometers placed in shoe insoles [32]. These methods, however, were limited in the sense of placing multiple sensors on various parts of the body, taking a long period of time to gather data, or showing insufficient performance of identification. The list of related studies is summarized in Table 1. Except for [8, 32], all the related studies collected data using only IMU sensors. On the other hand, we collected the data using insoles, where both IMU sensors and pressure sensors are installed within them. The distinguishing feature of our research from related studies is the use of pressure sensors with IMU sensors. Note that the pressure sensor data are useful due to the fact that not only they have not been taken into account in the other studies, but also the time series is split into small fragments corresponding to the phase of human gait. The detailed description is presented in Section 3.

**Table 1:** List of the related work on gait recognition.

Authors	Year	Sensor position	Sensor types
Luo et al. [33]	2020	Trunk, wrist, thighs, shanks	Acceleration, gyroscope, magnetic, orientation
Moon et al. [8]	2020	Foot	Pressure, acceleration, gyroscope
Choi et al. [32]	2019	Foot	Pressure, acceleration
Weiss et al. [34]	2019	Pants pocket, hand	Acceleration, gyroscope
Gadaleta et al. [30]	2018	Pants pocket	Acceleration, gyroscope
Al Kork et al. [35]	2017	Upper pocket, wrist, pants pocket, bag, leg, hand	Acceleration, gyroscope
Chereshnev et al. [36]	2017	Foot, shanks, thighs	Acceleration, gyroscope
Subramanian et al. [37]	2015	Pocket, holster	Acceleration, gyroscope, magnetic, orientation
Ngo et al. [38]	2014	Inside backpack	Acceleration, orientation
Anhuita et al. [39]	2013	Waist	Acceleration, gyroscope
Frank et al. [40]	2013	Pocket	Acceleration
Reiss et al. [41]	2012	Chest, wrist, ankle	Acceleration, gyroscope, magnetic
Zhang et al. [42]	2012	Hip	Acceleration
Altun et al. [43]	2010	Knees, chest, wrists	Acceleration, gyroscope, magnetic
Bächlin et al. [44]	2010	Shank, thigh, lower back	Acceleration
Gafurov et al. [45]	2010	Ankle	Acceleration

As for an open set gait recognition problem, a few studies have been conducted, each of which was performed differently. For example, a CNN-based classification model was designed to have a softmax output layer including a ‘not recognized’ class for unknown subjects at the time of training [46]. However, this approach is not scalable since the network model needs to be trained whenever a new subject is added to the system. In another study [30], a framework based on CNN and OSVM was used, but the system requires about a hundred unit steps to train the OSVM while not being evaluated with samples of truly unknown subjects. More recently, the open set problem was successfully handled in [47], proposing a framework with an ensemble model of CNN and recurrent neural network (RNN) along with the OSVM algorithm. It requires only a few unit steps to train OSVM while being evaluated with unseen samples of both known and unknown subjects to the OSVM-based classifier.

### 2.2 Explainable neural networks

Although a wide range of studies on XAI have been carried out in various domains [48–50], *post-hoc* methods, operating on the underlying model to be interpreted after the training has ended, have received considerable attention due to ease of implementation, as in our study. As one of popular post-hoc methods, SA measures the gradient values from the output to the input [19]. As another post-hoc method, LRP redistributes the scores of the output layer back to the input, rather than the gradient values [20]; this relies on the activation values during the feedforward process to determine how the values of each layer should be distributed. Other XAI methods have also been developed. Based on two axioms for attribution maps, integrated gradients [51] calculates the average gradient while following a linear path from a baseline (usually having the zero input). DeepLIFT [52] extended the LRP method by taking into account the activation of the baseline as a reference. Besides, in [53], the LRP method was applied to predict the category of text documents using standard machine learning models such as CNN.

To evaluate attribution maps, region perturbation was introduced in [54], where occluding parts of the input are shown with respect to relevance scores. According to the method in [54], the occluded parts were replaced by randomly sampled values. As an alternative, those parts were substituted with zero values [55]. In our study, we make some modifications in such a way that the absolute values of the relevance scores are used.

## 3 Data description and prototyping

**Fig 2:**
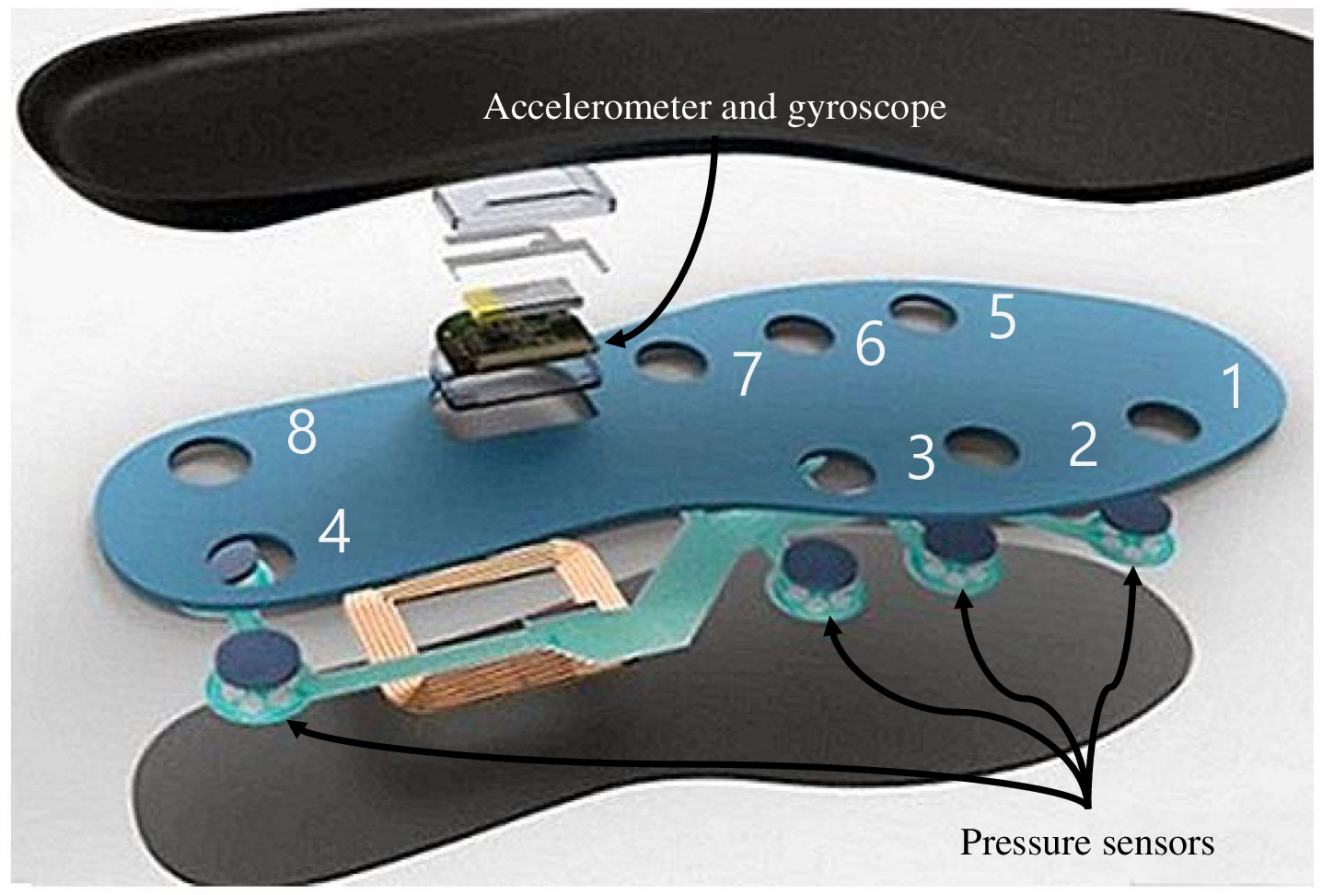
The insole used to collect the gait data.

To collect the gait information of the subjects, we utilized a commercial shoe insole, FootLogger [56], as illustrated in Fig 2. Eight pressure sensors, one 3D-axis accelerometer, and one 3D-axis gyroscope were installed in the insole. The pressure sensor gauges the pressure at one of three levels, and the accelerometer as well as the gyroscope measure the acceleration and rotation, respectively, leading to 2^16^ levels. While the subjects walked, the insole recorded data at every 0.01 second.

The pressure sensors report non-zero values when a foot is on the ground and show zero values otherwise. Using this property, as in [8], the original time series is converted into a series of unit steps in a fixed length, each of which includes data for one walking cycle. We partially adapted the notation in [47]. The *i*^th^ data of subject *id* = *a* for sensing modality *m* are denoted by ${\text{s}}_{i,a}^{m}$, where $m\in M=\{p_{l1},\cdots ,p_{l8},p_{r1},\cdots ,p_{r8},a_{lx},\cdots ,a_{rz},r_{lx},\cdots ,r_{rz}\}$. M is a set of all modalities. For example, *p*_*l*1_ denotes the pressure from the pressure sensor *id* = 1 in the left foot insole; *a*_*lx*_ denotes the acceleration along the *x*-axis in the left foot insole; and *r*_*rz*_ denotes the rotation along the *z*-axis in the right foot insole. We define ${\text{s}}_{i,a}^{pre}$, ${\text{s}}_{i,a}^{acc}$, ${\text{s}}_{i,a}^{rot}$, and **s**_*i*,*a*_ as follows:

$$\begin{array}{c} \begin{array}{cc} {\text{s}}_{i,a}^{pre} & =[{\text{s}}_{i,a}^{p_{l1}},\cdots ,{\text{s}}_{i,a}^{p_{l8}},{\text{s}}_{i,a}^{p_{r1}},\cdots ,{\text{s}}_{i,a}^{p_{r8}}]\\ {\text{s}}_{i,a}^{acc} & =[{\text{s}}_{i,a}^{a_{lx}},{\text{s}}_{i,a}^{a_{ly}},{\text{s}}_{i,a}^{a_{lz}},{\text{s}}_{i,a}^{a_{rx}},{\text{s}}_{i,a}^{a_{ry}},{\text{s}}_{i,a}^{a_{rz}}]\\ {\text{s}}_{i,a}^{rot} & =[{\text{s}}_{i,a}^{r_{lx}},{\text{s}}_{i,a}^{r_{ly}},{\text{s}}_{i,a}^{r_{lz}},{\text{s}}_{i,a}^{r_{rx}},{\text{s}}_{i,a}^{r_{ry}},{\text{s}}_{i,a}^{r_{rz}}]\\ {\text{s}}_{i,a} & =[{\text{s}}_{i,a}^{pre},{\text{s}}_{i,a}^{acc},{\text{s}}_{i,a}^{rot}]. \end{array} \end{array}$$

We call **s**_*i*,*a*_ by the *i*^th^ *unit step* of subject *id* = *a*. In addition, for sensing modality *m* and subject *id* = *a*, the *prototype* is defined as follows:

$$\begin{array}{c} {\text{c}}_{a}^{m}=\frac{1}{q}\sum_{i=1}^{q}{\text{s}}_{i,a}^{m}, \end{array}$$ (1)

where *q* is the number of unit steps of subject *id* = *a*. Then, we define the prototypes for three types of sensors by

$$\begin{array}{c} \begin{array}{cc} {\text{c}}_{a}^{pre} & =[{\text{c}}_{a}^{p_{l1}},\cdots ,{\text{c}}_{a}^{p_{l8}},{\text{c}}_{a}^{p_{r1}},\cdots ,{\text{c}}_{a}^{p_{r8}}]\\ {\text{c}}_{a}^{acc} & =[{\text{c}}_{a}^{a_{lx}},{\text{c}}_{a}^{a_{ly}},{\text{c}}_{a}^{a_{lz}},{\text{c}}_{a}^{a_{rx}},{\text{c}}_{a}^{a_{ry}},{\text{c}}_{a}^{a_{rz}}]\\ {\text{c}}_{a}^{rot} & =[{\text{c}}_{a}^{r_{lx}},{\text{c}}_{a}^{r_{ly}},{\text{c}}_{a}^{r_{lz}},{\text{c}}_{a}^{r_{rx}},{\text{c}}_{a}^{r_{ry}},{\text{c}}_{a}^{r_{rz}}]. \end{array} \end{array}$$

**Fig 3:**
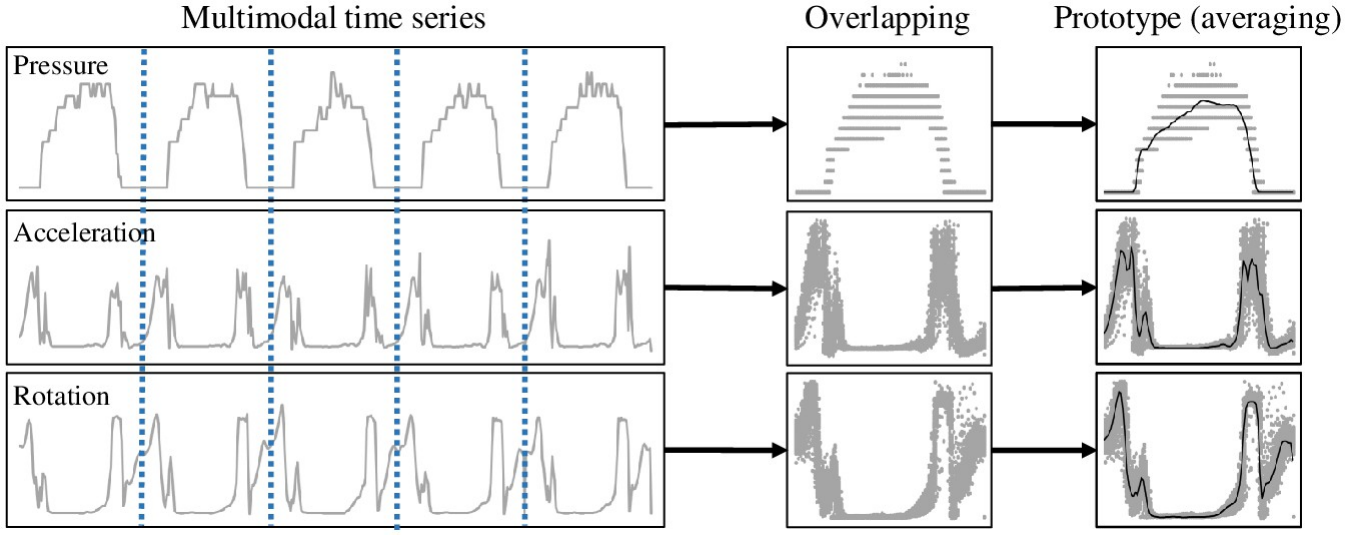
Illustration of computing the prototype of each sensing modality for a subject. The prototypes (bold solid curves in the rightmost figures) of a subject are computed by averaging over all unit steps. For brevity, the *L*_2_ norms of $s_{i,a}^{pre},s_{i,a}^{acc},\;\text{and}\;s_{i,a}^{rot}$ are depicted.

A conceptual diagram of computing the prototype is depicted in Fig 3.

## 4 Proposed methods

We assume a wireless environment in which all participants wear shoes with sensor-equipped insoles that can communicate wirelessly. The research problems are stated as follows:

- Given a set of data collected using the insoles, we aim at recognizing users using our encoder–decoder network to be more robust and less dependent on the values of hyper-parameters;
- Given the trained network for the gait recognition, we aim at demonstrating how high and low relevant parts of the data affect the accuracy of the recognition.

We first present our encoder–decoder architecture for gait recognition in Subsection 4.1. We then elaborate on two types of XAI methods built upon the designed architecture in Subsection 4.2.

The study was conducted according to the guidelines of the Declaration of Helsinki and approved by the Institutional Review Board of California State University Long Beach (IRB No. 21-091).

### 4.1 Gait recognition

In this subsection, we describe our proposed encoder–decoder network architecture and a few-shot learning approach for gait recognition. In the previous work [47], an ensemble model of CNN and RNN with the triplet loss function showed the recognition accuracy about 93%, which is however quite sensitive to the values of hyper-parameters. This motivates us to propose the encode–decoder network with the combination of the prototype and triplet loss function to overcome the issue.

#### 4.1.1 Network architecture

**Fig 4:**
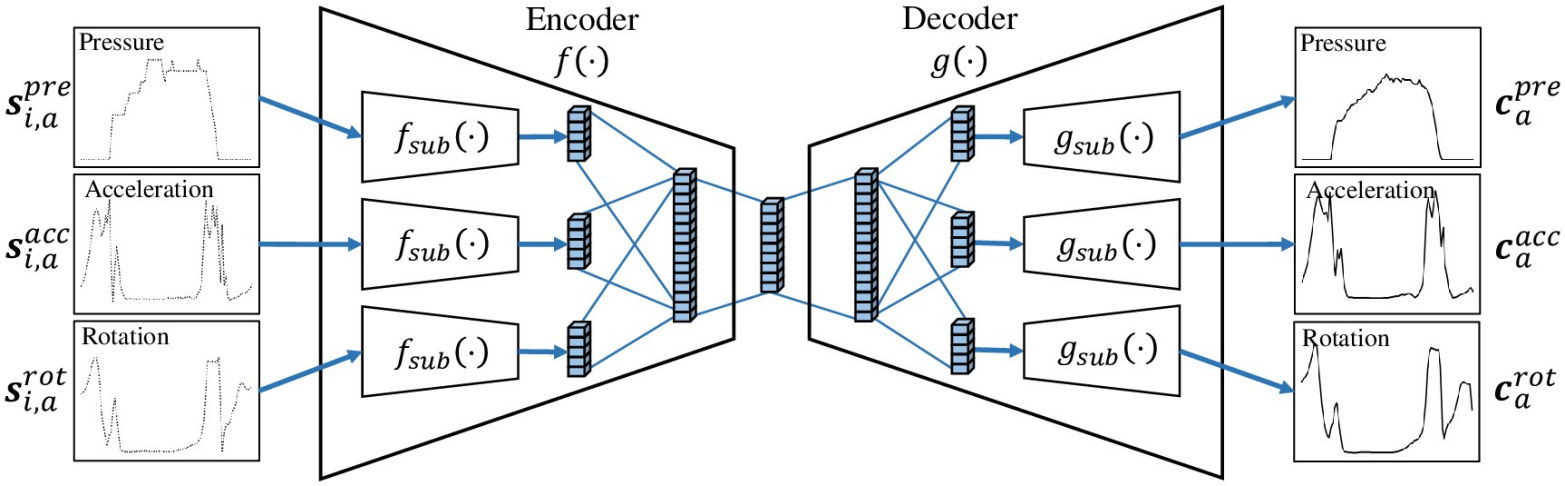
Illustration of the encoder–decoder architecture. The encoder and decoder include three sub-encoders and sub-decoders for multimodal sensing.

We propose an encoder–decoder network architecture alongside two loss functions. The encoder *f*(⋅) maps a unit step to an embedding vector in a latent space, and the decoder *g*(⋅) maps an embedding vector to a prototype. The encoder *f*(⋅) includes three identical sub-encoders *f*_*sub*_(⋅), which consist of three one-dimensional (1D) convolutional layers with 32, 64, and 128 filters and a flattened layer in order. The last flattened layers of three sub-encoders are fully connected to a dense layer with 256 units, and the dense layer is fully connected to another dense layer with 128 units, which is the output of the encoder. Similarly as in the encoder, the decoder *g*(⋅) includes one dense layer with 256 units and three identical sub-decoders *g*_*sub*_(⋅), which consist of the same layers as those in the sub-encoders in reverse order. Although the layouts of the sub-encoders or sub-decoders are identical, their parameters are independently trained using different sensing modalities, including pressure, acceleration, and rotation. The network architecture is depicted in Fig 4.

In the encoder–decoder network architecture, we take the middle dense layer with 128 units as the output of the encoder. The encoder maps unit steps of $\text{s}≜[{\text{s}}_{i,a}^{pre},{\text{s}}_{i,a}^{acc},{\text{s}}_{i,a}^{rot}]$ to embedding vectors **v**:

$$\begin{array}{c} f\left({\text{s}}_{i,a}^{pre},{\text{s}}_{i,a}^{acc},{\text{s}}_{i,a}^{rot}\right)=f\left(\text{s}\right)=\text{v}, \end{array}$$ (2)

where the dimension of embedding vectors is 128 (i.e., $f\left(\cdot \right)\in R^{128}$) and the vectors are normalized to 1 (i.e., ||*f*(⋅)||_2_ = 1). Hereafter, to simplify notations, ${\text{s}}_{i,a}^{pre}$, ${\text{s}}_{i,a}^{acc}$, and ${\text{s}}_{i,a}^{rot}$ will be written as **s**^*pre*^, **s**^*acc*^, and **s**^*rot*^, respectively, if dropping the subscript (*i*, *a*) does not cause any confusion.

Let **s**_*i*,*a*_ and **s**_*j*,*a*_ (*i* ≠ *j*) be two unit steps of subject *id* = *a*, and let **s**_*k*,*b*_ be a unit step of subject *id* = *b*. Similarly as in the triplet loss [11], our multimodal triplet loss is defined as follows:

$$\begin{array}{c} L_{triplet}=\left|\right|{\text{v}}_{i,a}-{\text{v}}_{j,a}{\left|\right|}_{2}^{2}-\left|\right|{\text{v}}_{i,a}-{\text{v}}_{k,b}{\left|\right|}_{2}^{2}+\alpha , \end{array}$$ (5)

where **v**_*i*,*a*_ = *f*(**s**_*i*,*a*_), **v**_*j*,*a*_ = *f*(**s**_*j*,*a*_), **v**_*k*,*b*_ = *f*(**s**_*k*,*b*_), and *α* ≥ 0 is a margin. We set *α* = 1.25 in the experiments unless otherwise stated.

The multimodal triplet loss attempts to ensure that the distance between two embedding vectors **v**_*i*,*a*_ and **v**_*j*,*a*_ is smaller than the distance between another pair of embedding vectors **v**_*i*,*a*_ and **v**_*k*,*b*_ for all possible triplets in the *training* set. However, an encoder with the triplet loss function can be severely distorted when the variation of the unit steps of one subject is large. To alleviate this drawback of the traditional triplet loss function, we propose an encoder–decoder architecture with a *prototyping loss*. This is similar to the denoising autoencoders [12], the center loss encoder [57], or the variational prototyping encoder [58]; however, the proposed method does not corrupt the original data, does not compute the loss in a latent space, and does not require additional prototypes as input. For each sensing modality m∈M, the prototyping loss is defined as follows:

$$\begin{array}{c} L_{proto}=\frac{1}{\left|M\right|}\sum_{m\in M}||g\left(f\left({\text{s}}_{i,a}^{m}\right)\right)-{\text{c}}_{a}^{m}{\left|\right|}_{2}^{2}. \end{array}$$ (4)

**Fig 5:**
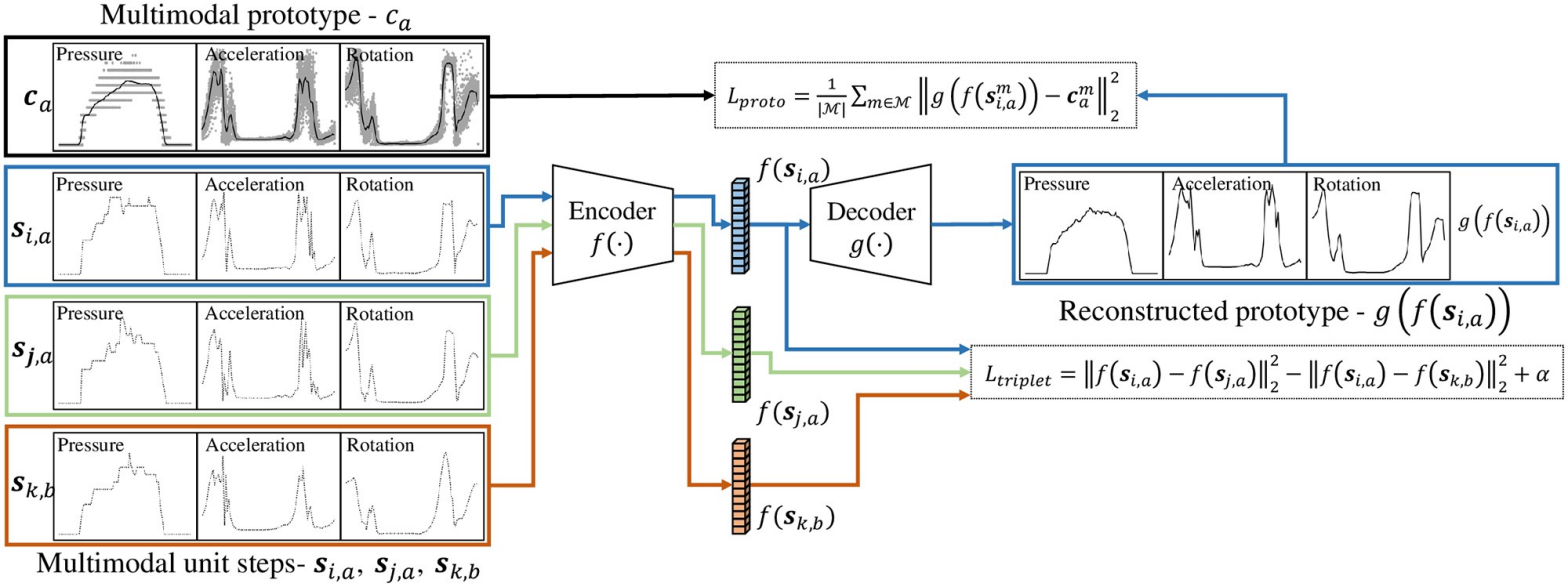
Illustration of the prototyping encoder–decoder with triplet loss. The overall loss function is a linear combination of the multimodal triplet loss function and the prototyping loss function.

Before computing the loss above, both $g(f\left({\text{s}}_{i,a}^{m}\right))$ and ${\text{c}}_{i}^{m}$ are normalized to 1, that is $||g\left(f\left({\text{s}}_{i,a}^{m}\right)\right){\left|\right|}_{2}=\left|\right|{\text{c}}_{i}^{m}{\left|\right|}_{2}=1$. Since the overall loss function is given by a linear combination of the multimodal triplet loss function and the prototyping loss function, it is formulated as

$$\begin{array}{c} L=L_{triplet}+\lambda L_{proto}, \end{array}$$ (5)

where λ ≥ 0 is one of the hyper-parameters of the system. A conceptual diagram of the prototyping encoder–decoder architecture is illustrated in Fig 5.

#### 4.1.2 Few-shot learning

We split the subjects into the following three groups: *training*, *known*, and *unknown* groups. For the *training* group, all unit steps of the subjects are allocated to the *training* set. For the *known* group, *n* unit steps are utilized to compute the centroids of the embedding vectors and to learn the decision boundaries of the subjects using the OSVM algorithm [13], where *n* is one of hyper-parameters of the system, and we set *n* = 10 in the experiments unless otherwise stated. Subsequently, all unit steps with the exception of *n* unit steps in the *known* group are allocated to the *known test* set. For the *unknown* group, all unit steps are allocated to the *unknown test* set. From now on, we design our method based on few-shot learning (see [47] and references therein).

**Fig 6:**
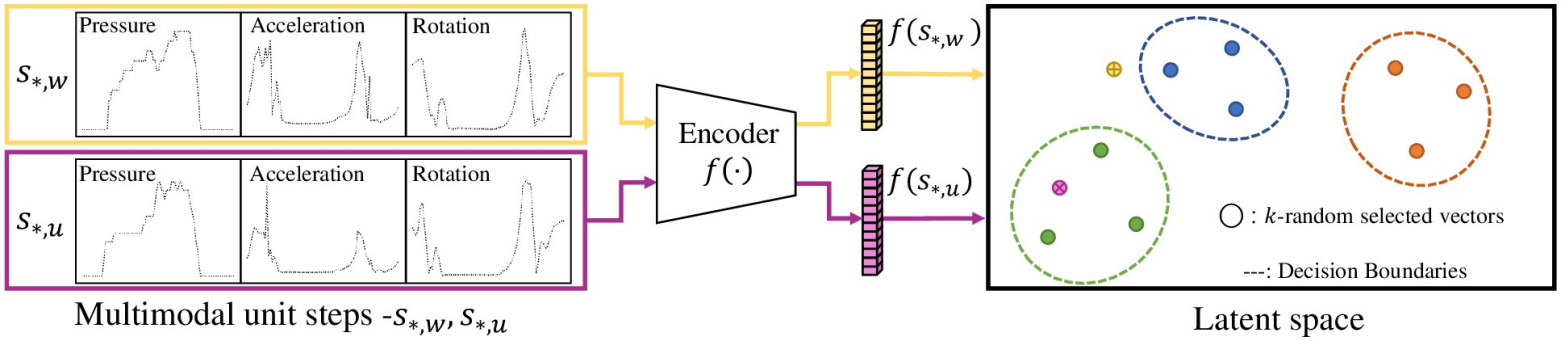
Illustration of gait recognition using the trained encoder. Here, unit step s_*,*u*_ is recognized as that of the “green” subject, whereas unit step s_*,*w*_ is rejected.

Let s_*,*u*_ be a unit step of subject *id* = *u* in either the *known test* or *unknown test* set. The symbol * indicates that the unit step can be any unit step of subject *u*. To recognize s_*,*u*_, the system first computes v_*,*u*_ = *f*(s_*,*u*_) and finds the provisional subject *id* = *p* such that $p=\text{arg}\;{\text{min}}_{a}\left|\right|{\text{D}}_{a}-{\text{v}}_{*,u}{\left|\right|}_{2}^{2}$, where ${\text{D}}_{a}=\frac{1}{n}\sum_{i=1}^{n}{\text{v}}_{i,a}$ for all subjects *a* in the *known* group. Here, the centroid **D**_*a*_ is computed using *n* unit steps, which are not included in the *known test* set. Next, the system discovers a decision boundary of subject *p* using the OSVM algorithm. Specifically, for the *n* embedding vectors of subject *p*, the algorithm uses the computed set {**v**_*i*,*p*_|1 ≤ *i* ≤ *n*} as input and then solves the following optimization problem:

$$\begin{array}{c} \left\{ \begin{array}{cc} & {\text{min}}_{\alpha }\frac{1}{2}\sum_{i}^{n}\sum_{i^{\prime }}^{n}\alpha_{i}\alpha_{i^{\prime }}K\left({\text{v}}_{i,p},{\text{v}}_{i^{\prime },p}\right)\\ & \text{subject}\;\text{to:}\;0\leq \alpha_{i}\leq \frac{1}{\nu n},\sum_{i=1}^{n}\alpha_{i}=1, \end{array}\right. \end{array}$$ (6)

where $K\left(\text{v},{\text{v}}^{\prime }\right)=e^{-\gamma ||\text{v}-{\text{v}}^{\prime }{\left|\right|}_{2}^{2}}$ is a radial bias kernel function; *α*_*i*_ are the Lagrange multipliers; and *γ* and *ν* are the hyper-parameters of the system. The decision function of v_*,*u*_ for subject *p* is defined by

$$\begin{array}{c} h_{p}\left({\text{v}}_{*,u}\right)=\sum_{i}^{n}\alpha_{i}K\left({\text{v}}_{i,p},{\text{v}}_{*,u}\right)-\delta_{p}, \end{array}$$ (7)

where $\delta_{p}=\sum_{i}^{n}\alpha_{i}K\left({\text{v}}_{i,p},{\text{v}}_{h,p}\right)$ for any *h* that fulfills the condition $0<\alpha_{h}<\frac{1}{\nu n}$ and 1 ≤ *h* ≤ *n*. Finally, the system recognizes subject *u* as subject *p* if *h*_*p*_(v_*,*u*_) ≥ *τ*, where *τ* is one of the hyper-parameters of the system. A conceptual diagram of the few-shot learning-based recognition is illustrated in Fig 6, and the detailed procedure is summarized as follows:

1. Compute v_*,*u*_ = *f*(s_*,*u*_);
2. Find a provisional subject $p=\text{arg}\;{\text{min}}_{a}\left|\right|{\text{D}}_{a}-{\text{v}}_{*,u}{\left|\right|}_{2}^{2}$;
3. If *h*_*p*_(v_*,*u*_) ≥ *τ*, then “*u* is recognized as *p*”.
4. Otherwise, “*u* is not recognized”

### 4.2 Explainable gait recognition

We describe two types of XAI methods for gait recognition built upon our encoder–decoder network architecture.

#### 4.2.1 Methods for explanation

After we train the encoder *f*(⋅), we further implement two types of XAI methods, including SA and LRP, to gain transparency in our model and understand the decision process [14]. That is, we aim to explain our encoder *f*(⋅) by generating attribution maps that have the same dimensions as those in the input. Ideally, the values in an attribution map represent the importance (also known as the *relevance score*) of the input in the same position when the encoder calculates the embedding vector. For the encoder *f*(⋅) and given unit step input **s** = [**s**^*pre*^, **s**^*acc*^, **s**^*rot*^], our objective is to calculate an attribution map $A_{c}\left(\text{s}\right)$, where ${\left(A_{c}\left(\text{s}\right)\right)}_{ij}$ captures the degree to which the *c*-th component of *f*(**s**) is relevant to the *i*-th row and *j*-th column in the input **s**. Next, we would like to describe two XAI methods for gait recognition as in the following.

*4.2.1.1 Sensitivity analysis (SA)*. One of the most widely adopted methods is to use the gradient that flows from the output to the input [19]. The gradient ∇_**s**_(*f*(**s**))_*c*_ can be interpreted as measuring the sensitivity of the input value affecting the *c*-th component of the output. That is, high gradient values indicate that small deviations in the input can result in substantial changes in the model output. Each component of the gradient-based attribution map is defined as follows:

$$\begin{array}{c} {\left(A_{c}\left(\text{s}\right)\right)}_{ij}=|\frac{\partial {\left(f\left(\text{s}\right)\right)}_{c}}{\partial {\left(\text{s}\right)}_{ij}}|. \end{array}$$ (8)

The gradient attribution map can be efficiently calculated using a back-propagation algorithm without any re-training of the encoder.

*4.2.1.2 Layer-wise Relevance Propagation (LRP)*. We also take into account LRP [20] to interpret our gait recognition encoder. This method also does not require additional training of the model and efficiently calculates attribution maps. LRP calculates the relevance attribution map of given input **s** by redistributing the output value of the encoder *f*(⋅) back to the input. LRP starts by defining the relevance score of the final layer as the value of the output layer itself. Then, from the output layer, the method redistributes the relevance score through each layer in an iterative manner, while computing the relevance scores for each hidden layer. In general, let us denote each layer in the encoder *f*(⋅) as {*l*^(0)^, ⋯, *l*^(*L*−1)^, *l*^(*L*)^} in a sequential manner, where *l*^(0)^ is the input layer and *l*^(*L*)^ is the output layer. We also define the relevance score of neuron *k* in layer *l*^(*H*)^ as $R_{k}^{\left(H\right)}$. As mentioned before, we define the relevance score in the final layer *l*^(*L*)^ as the output value of the model itself, i.e., $R_{k}^{\left(L\right)}={\left(f\left(\text{s}\right)\right)}_{k}$. To describe how the output value is redistributed back to the input layer, we consider an intermediate or the output layer *l*^(*h*)^ for *h* = 1, ⋯, *L*. For all neurons *j* in layer *l*^(*h*)^, the relevance score $R_{j}^{\left(h\right)}$ is redistributed to the neuron *i* in the previous layer *l*^(*h*−1)^ through the following redistribution rule:

$$\begin{array}{c} R_{i}^{\left(h-1\right)}=\sum_{j}\frac{x_{i}^{\left(h-1\right)}w_{ij}^{\left(h-1,h\right)}}{\sum_{i}x_{i}^{\left(h-1\right)}w_{ij}^{\left(h-1,h\right)}}R_{j}^{\left(h\right)}, \end{array}$$ (9)

where $x_{i}^{\left(h-1\right)}$ is the activation value of the *i*-th neuron in layer *l*^(*h*−1)^ and $w_{ij}^{\left(h-1,h\right)}$ is the trained weight between neuron *i* in layer *l*^(*h*−1)^ and neuron *j* in layer *l*^(*h*)^. The redistribution rule is applied until it reaches the input layer, which becomes $A_{c}\left({\text{s}}_{*,a}\right)$. A modified version of LRP, dubbed LRP-*ϵ*, is frequently used, which adds a small stabilizer term *ϵ* in the denominator of Eq (9). The role of *ϵ* is to absorb some weak or contradictory relevance, thereby leading to sparser and less noisy descriptions [59]. We adopt this LRP-*ϵ* method in our experiments and use the iNNvestigate toolbox [60] to calculate the attribution maps.

#### 4.2.2 Attribution maps for open set gait recognition

Typically, methods generating attribution maps are applied to neural networks for performing classification tasks [19, 20, 55], which is applicable to the closed set recognition. In this case, each component in the output is interpreted as the inferred probability. In this context, the attribution map $A_{c}$ represents the relevance scores of the input to the probability that the input is classified as class *c*.

However, in the open set recognition setting, the encoder returns embedding vectors for each unit step **s**, rather than a vector of the probabilities. Due to the fact that the components in the output do not have explicit meaning, it may be hardly possible to directly apply the previous approach for interpretation. In our study, we propose another strategy that averages out the attribution maps over all 128 components in the embedding vectors to see how the trained encoder views the input s_*,*a*_. Formally, the attribution map is defined as

$$\begin{array}{c} A\left(\text{s}\right)=\frac{1}{128}\sum_{c=1}^{128}A_{c}\left(\text{s}\right). \end{array}$$ (10)

## 5 Evaluation and results

In this section, we first describe the experimental settings and evaluation metrics. Next, we present evaluation results for the proposed gait recognition with the prototyping encoder–decoder architecture. Finally, we comprehensively demonstrate the evaluation results of our XAI methods using attribution maps.

### 5.1 Experimental settings and evaluation metrics

It is worth noting that one of the key component of the proposed method is utilization of the pressure data. To have pressure data while walking, we collected gait information data by ourselves using the insole from 40 adults as they walked for approximately 3 minutes. The entire dataset consists of 6,303 unit steps, which correspond to 158 unit steps per subject. We also note that, although a much higher number of subjects might be utilized from publicly available datasets (e.g., [6, 7]), we do not adopt such datasets in our experiments since they basically lack pressure sensors that play a crucial role in our study.

To see the impact of our occlusion strategy that replaces the corresponding parts by zero, the original data with pressure sensors of 0’s are first replaced by *δ* through data pre-processing. The *δ* was set to 0.01 to make minimal adjustments to the original data.

**Fig 7:**
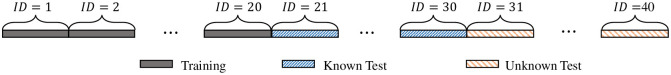
Illustration of splitting the data into the *training, known test*, and *unknown test* sets.

As shown in Fig 7, the data were split into three sets: *training*, *known test*, and *unknown test* sets. First, we sampled 20 subjects from 40 subjects, and all of their unit steps were assigned to the *training* set for the encoder–decoder network. Second, the other 20 subjects were divided into two groups equally. 10 unit steps of 10 subjects in one group were used to train the OSVM classifier, and the remaining unit steps of the subjects in the group were kept for the *known test* set. Finally, 100% of the unit steps of 10 subjects in the other group were allocated to the *unknown test* set. The *known test*, *unknown test*, and *training* sets contain approximately 1,480, 1,580, and 3,160 unit steps, respectively. Such datasets were generated 10 times repeatedly.

We trained and evaluated the network with each dataset. We then evaluated the performance metrics averaged from 10 repetitions. When a unit step in the *known test* set is recognized correctly, we define such an event as a *true positive* (TP), otherwise a *false negative* (FN). In contrast, if a unit step in the *unknown test* set is not recognized as any subject known, we define such an event as a *true negative* (TN) or otherwise a *false positive* (FP). Subsequently, we denote the true positive rate as $TPR=\frac{TP}{TP+FN}$, the true negative rate as $TNR=\frac{TN}{TN+FP}$, and the accuracy as $ACC=\frac{TP+TN}{TP+FN+TN+FP}$.

### 5.2 Evaluation for gait recognition

**Fig 8:**
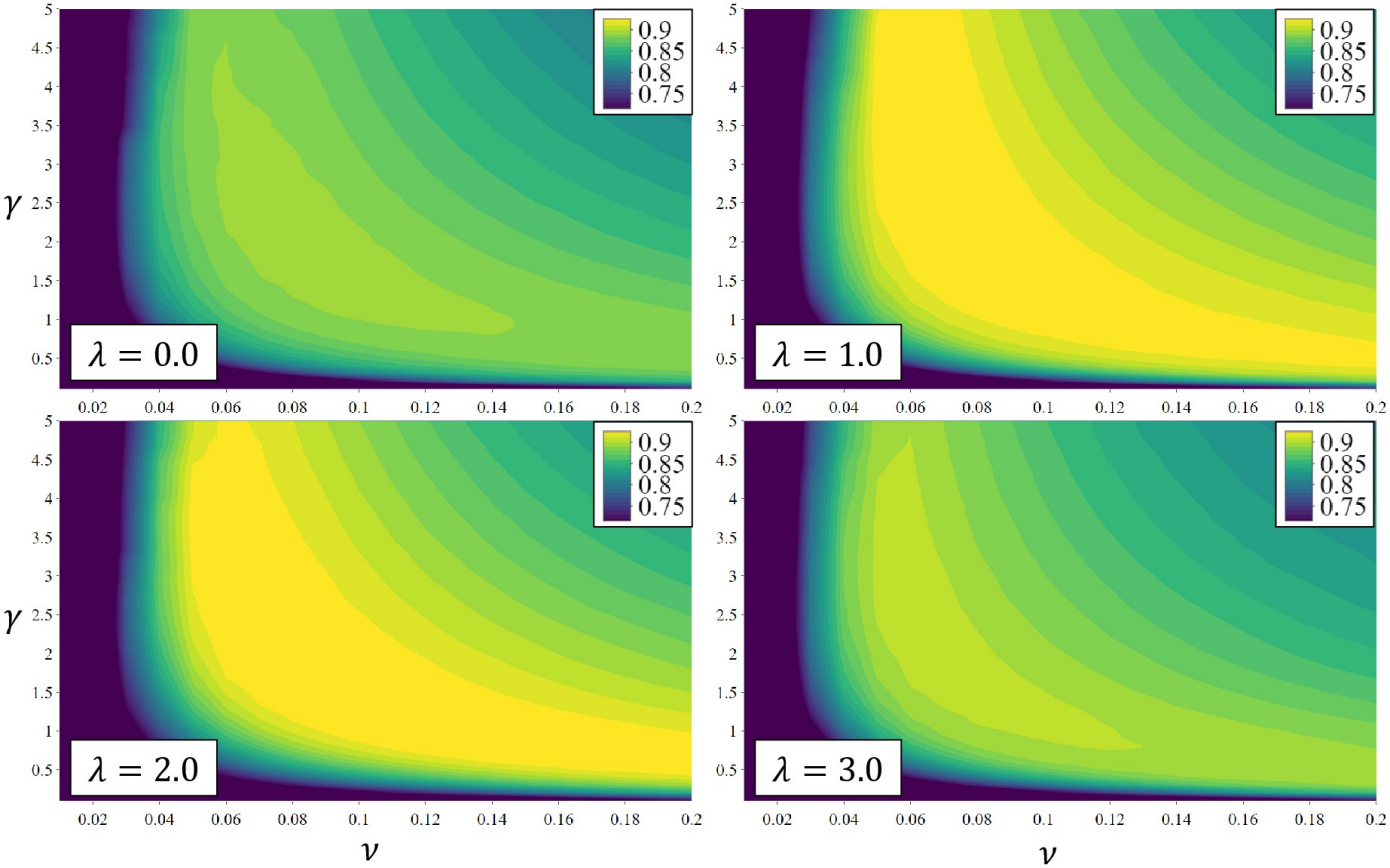
ACC as a function of *γ* and *ν* for a value of *τ* = −0.1. A similar rate is represented as the same color with the maximum 1% difference, with the highest rates as yellow.

The distributions of ACC as a function of hyper-parameters *γ* and *ν* for different values of λ are shown in Fig 8. Clearly, selection of *γ* and *ν* is critical to the overall recognition accuracy of our model. The area in which the rates are greater than 90% (corresponding to the yellow area) indicates that the region for λ = 1.0 is broader than the regions for other cases. This means that the case of λ = 1.0 has a weak dependency when *γ* and *ν* are selected, which affects the robustness to the recognition performance. In practice, the hyper-parameters need to be tuned by considering both the TPR and TNR simultaneously. For example, if all unit steps are rejected, then we could achieve the TNR of 100% at the cost of 0% of TPR. Thus, we set the hyper-parameters in the sense of minimizing the difference between the TPR and TNR.

**Fig 9:**
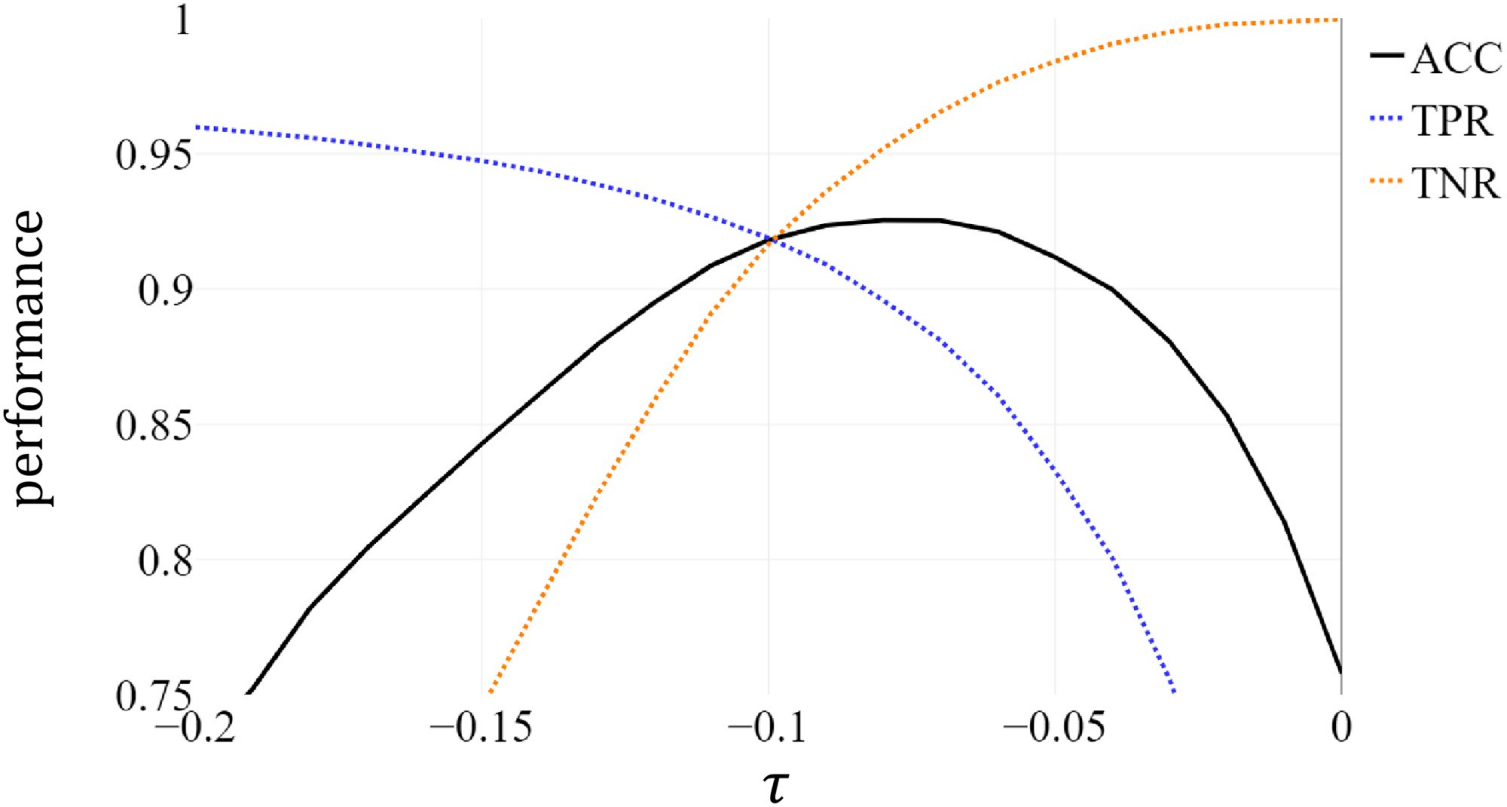
Performance as a function of *τ* for fixed values of *γ* = 2.2, *ν* = 0.06, and λ = 1.0.

To examine the effect of *τ*, we used *γ* = 2.2 and *ν* = 0.06 for λ = 1.0. In Fig 9, we can see that the TPR and ACC get considerably enhanced when *τ* is smaller than 0. Based on this observation, it is desirable to choose alternative *τ* instead of *τ* = 0.0 for the decision boundary in the latent space.

### 5.3 Evaluation for attribution maps

#### 5.3.1 Extraction of common attribution maps

In our study, we would like to answer the following question: *can we identify what parts of the unit steps are the most relevant to the open set gait recognition, regardless of the subject id?* In other words, we aim to find an attribution map where the relevance values are commonly valid for most of the unit steps. To achieve this goal, we define a *common attribution map* $A_{com}$ as the attribution map averaged out over all unit steps in the *training* set:

$$\begin{array}{c} A_{com}=\frac{1}{\sum_{a}^{m}n_{a}}\sum_{a}^{m}\sum_{i}^{n_{a}}A\left({\text{s}}_{i,a}\right), \end{array}$$ (11)

where *a* denotes the subject index, *i* denotes the unit step index, *m* denotes the number of subjects in the *training* set, and *n*_*a*_ denotes the number of unit steps of the subject *id* = *a* in the *training* set.

#### 5.3.2 Evaluation methods

As in [54, 55], we choose *region perturbation* to evaluate the common attribution map from Eq (11) with some modifications. For the given unit step **s** and the common attribution map $A_{com}$ (derived from either SA or LRP-*ϵ*), it is possible to order the relevance scores in $A_{com}$ from the highest to the lowest. The region perturbation observes the amount of performance degradation when specific parts of the unit step are occluded (i.e., replaced by zero). The intuition behind is that, if we occlude regions of the unit step with high relevance, then the performance degradation will be significant compared to the case in which we occlude regions with low relevance.

For the evaluation, we consider a sequence, denoted as *O* = (*pos*_1_, ⋯, *pos*_*i*_, *pos*_*j*_, ⋯, *pos*_*L*_), where *pos*_*i*_ indicates the position (e.g., row and column indices) and *L* is the total number of components in the unit step **s**. The sequence order is determined by the relevance score of the position, where, for two neighboring indices *i* and *j*, the attribution map scores for *pos*_*i*_ are greater than (or equal to) *pos*_*j*_. Hence, *pos*_1_ indicates the position in the unit step with the highest relevance score, and *pos*_*L*_ indicates the position of the lowest relevance score. As explained in Section 4.2.2, if the output of the network to be explained represents the classification probability, then each output component eventually responds to a binary (yes/no) question. In this case, the sign of the relevance scores can be interpreted as positively/negatively affecting a certain class. However, in our experiment, because each component of the embedding vectors (corresponding to the output of the encoder *f*(⋅)) has no explicit meaning, we use the *magnitude* of the relevance score to acquire the sequence *O*.

#### 5.3.3 Evaluation results for common attribution maps

To validate the effectiveness of common attribution maps, we first divide the sequence *O* into several groups. We equally divide *O* into five sub-sequences, *O*_1_, ⋯, *O*_5_. Thus, *O*_1_ includes the top 20% of the positions with the highest relevance score, and *O*_2_ includes the next 20% of the positions, and so on. We removed the positions in the unit step for *O*_1_, ⋯, *O*_5_ individually and then observed the metrics ACC, TPR, and TNR. We compare the results with a random baseline such that the same amount is occluded but the positions are randomly chosen.

**Fig 10:**
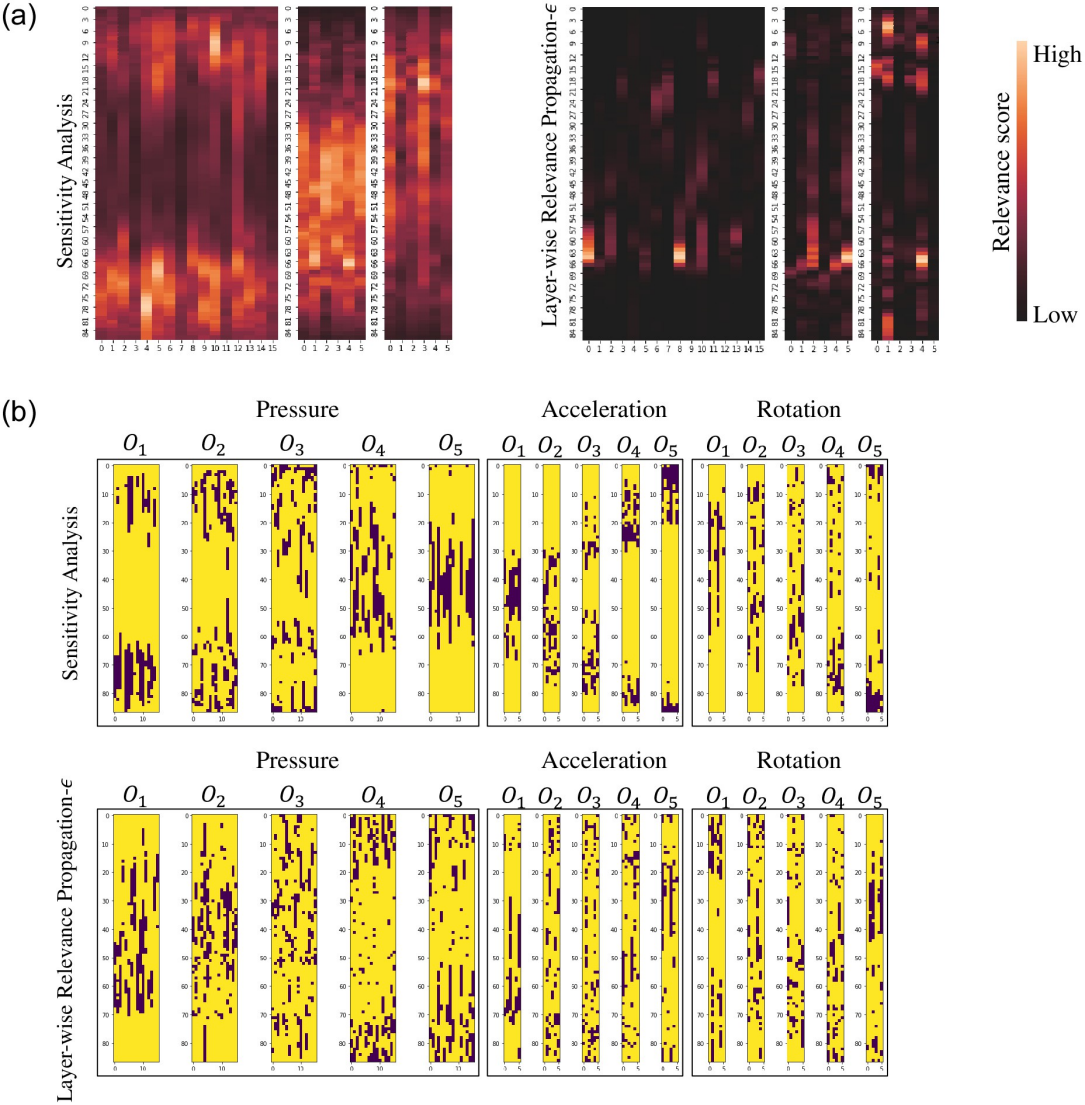
Averaged relevance score heat maps and the their occluding positions for *O*_1_ ⋯ *O*_5_ of SA and LRP-*ϵ*. In each heatmap, the x-axis and y-axis indicate features of each sensor and time-stamps of each unit step, respectively. (a) Common attribution maps. (Left: SA, Right: LRP-*ϵ*). (b) Occluding positions (*O*_1_, ⋯, *O*_5_) for all modal inputs. (Top: SA, Bottom: LRP-*ϵ*).

The heatmap visualizations and their occluding positions are shown in Fig 10a and 10b. As shown in Fig 10a, the attribution maps for both methods exhibit different patterns. For instance, in the pressure attribution maps, LRP-*ϵ* focuses on the parts of unit steps when feet are contacting with the ground (i.e., the stance phase). In contrast, SA focuses on the other parts of unit steps when feet are in the air (i.e., the swing phase). Subsequently, the occluding positions for *O*_1_, ⋯, *O*_5_ also reveal different patterns as depicted in Fig 10b.

**Fig 11:**
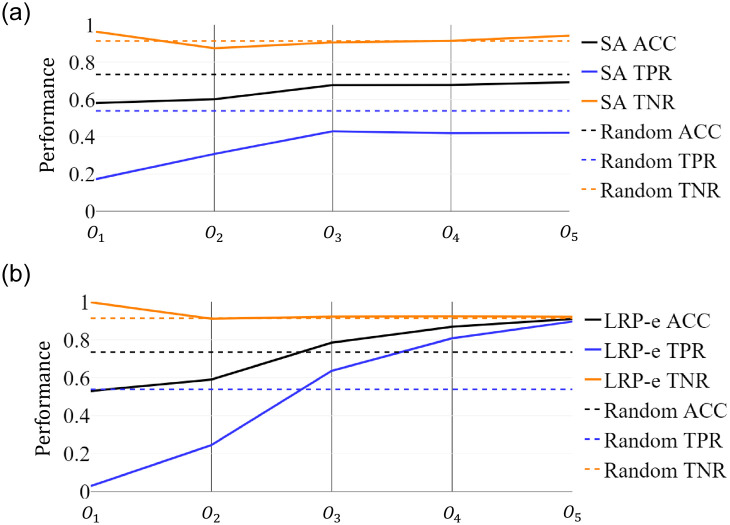
Performance as a function of occluding position *O*_1_, ⋯, *O*_5_ by SA and LRP-*ϵ* for fixed *γ* = 2.2, *ν* = 0.06, *τ* = −0.1, and λ = 1.0. (a) SA. (b) LRP-*ϵ*.

Furthermore, from Fig 11a and 11b, we can observe the overall performance degradation for both methods in terms of TPR and ACC as we move from occlusion of *O*_5_ to that of *O*_1_. The occlusion of *O*_1_ obtained from the attribution map results in TPR decrement of 0.3 and 0.5 for SA and LRP-*ϵ*, respectively, compared to the random baseline. This implies that both SA and LRP-*ϵ* can detect the most important unit step regions (*O*_1_) for the *known test* set. Especially, we observe that, if we occlude *O*_1_, then the TPR drops to zero for LRP-*ϵ*. When we pay our attention to less relevant occlusions, we see that the SA method exhibits a lower performance gain in TPR, resulting in inferior performance compared to the random baseline even in the case of *O*_5_. Meanwhile, LRP-*ϵ* surpasses the random baseline in *O*_3_ and results in much superior performance in *O*_5_. This demonstrates that using LRP-*ϵ* can offer more robust common attribution maps compared to SA. For both methods for *O*_1_, the TNR values are very close to 1 and the TPR values are very low. From these findings, we see that the model does not recognize almost every unit step. In consequence, both methods detect the most relevant part of the unit step while LRP-*ϵ* outperforms SA as seen from the ACC.

## 6 Concluding remarks

This paper presented a novel gait recognition system capable of revealing the most important parts of the multimodal time series to distinguish the individuals. The proposed encoder–decoder prototyping network architecture along with our loss functions successfully solved the open set gait recognition problem from the data collected using a wearable device. Our experiments demonstrated that the system’s recognition performance is less sensitive to the changes in the values of hyper-parameters than those in the previous studies. Furthermore, using XAI methods based on SA and LPR-*ϵ*, we provided insightful interpretability for the complex relations between the multimodal time series and the recognition results. The proposed common attribution map clearly revealed which part of the multimodal time series is relevant to the recognition performance.

Potential avenues of future research in this area include performance improvement of the end-to-end recognition system and design of more sophisticated XAI methods by optimizing the common attribution map.
